# Quantitative trait loci analysis of *Verticillium* wilt resistance in interspecific backcross populations of *Gossypium hirsutum* × *Gossypium barbadense*

**DOI:** 10.1186/s12864-016-3128-x

**Published:** 2016-11-05

**Authors:** Yuzhen Shi, Baocai Zhang, Aiying Liu, Wentan Li, Junwen Li, Quanwei Lu, Zhen Zhang, Shaoqi Li, Wankui Gong, Haihong Shang, Juwu Gong, Tingting Chen, Qun Ge, Tao Wang, Heqin Zhu, Zhi Liu, Youlu Yuan

**Affiliations:** 1 State Key Laboratory of Cotton Biology, Key Laboratory of Biological and Genetic Breeding of Cotton, The Ministry of Agriculture, Institute of Cotton Research, Chinese Academy of Agricultural Sciences, Anyang, 455000 Henan China; 2 College of Bioscience and Biotechnology, Hunan Agricultural University, Changsha, 410128 Hunan China

**Keywords:** Cotton, *Verticillium* wilt (VW), Quantitative trait loci (QTL), Interspecific backcross population

## Abstract

**Background:**

*Verticillium* wilt (VW) caused by *Verticillium dahliae* (*Kleb*) is one of the most destructive diseases of cotton. The identification of highly resistant QTLs or genes in the whole cotton genome is quite important for developing a VW-resistant variety and for further molecular design breeding.

**Results:**

In the present study, BC_1_F_1_, BC_1_S_1_, and BC_2_F_1_ populations derived from an interspecific backcross between the highly resistant line Hai1 (*Gossypium barbadense* L.) and the susceptible variety CCRI36 (*G. hirsutum* L.) as the recurrent parent were constructed. Quantitative trait loci (QTL) related to VW resistance were detected in the whole cotton genome using a high-density simple sequence repeat (SSR) genetic linkage map from the BC_1_F_1_ population, with 2292 loci covering 5115.16 centiMorgan (cM) of the cotton (AD) genome, and the data concerning VW resistance that were obtained from four dates of BC_2_F_1_ in the artificial disease nursery and one date of BC_1_S_1_ and BC_2_F_1_ in the field. A total of 48 QTLs for VW resistance were identified, and 37 of these QTLs had positive additive effects, which indicated that the *G. barbadense* alleles increased resistance to VW and decreased the disease index (DI) by about 2.2–10.7. These QTLs were located on 19 chromosomes, in which 33 in the A subgenome and 15 QTLs in the D subgenome. The 6 QTLs were found to be stable. The 6 QTLs were consistent with those identified previously, and another 42 were new, unreported QTLs, of which 31 QTLs were from *G. barbadense*. By meta-analysis, 17 QTL hotspot regions were identified and 10 of them were new, unreported hotspot regions. 29 QTLs in this paper were in 12 hotspot regions and were all from *G. barbadense*.

**Conclusions:**

These stable or consensus QTL regions warrant further investigation to better understand the genetics and molecular mechanisms underlying VW resistance. This study provides useful information for further comparative analysis and marker-assisted selection in the breeding of disease-resistant cotton. It may also lay an important foundation for gene cloning and further molecular design breeding for the entire cotton genome.

**Electronic supplementary material:**

The online version of this article (doi:10.1186/s12864-016-3128-x) contains supplementary material, which is available to authorized users.

## Background

Cotton is an important economic crop worldwide. It provides important natural fibers for the textile industry. Of the two most economically important tetraploid cultivated species, *Gossypium hirsutum* (Upland cotton), which has high yield and wide adaptability, makes up about 95 % of the total cotton yield worldwide [1]. *G. barbadense* (sea-island cotton), however, is grown in only limited areas because of its relatively low yield and limited adaptability, despite its strong resistance to *Verticillium* disease and extra-long, fine, strong, fibers. Cultivated forms of both these species show very different traits regarding yield, fiber quality, disease resistance, environmental adaptation, and other traits [2].

*Verticillium* wilt (VW) is one of the important diseases of cotton (*G. hirsutum* L.) worldwide. It is caused by *Verticillium dahliae* (*Kleb*.), a soil-borne fungal pathogen that has a broad range of hosts and moves among them using a variety of mechanisms. It has high pathogenicity and can survive for long periods [3]. Infected plants usually exhibit symptoms of marginal chlorosis or necrosis in leaves, discoloration of the stem vascular bundles, and even full defoliation and plant death. Severe infection results in significant reduction of fiber yield and quality [2, 4]. Worldwide, the disease was first reported in Virginia in 1914 [5]. Today, it is found in almost all cotton growing areas worldwide. This disease broke out in China in 1993, resulting in the infection of approximately 80 % of the cotton crop in the seriously infected region of cotton in North China and lint yield losses of 100 million kg in the whole China in that year [6, 7]. Another VW outbreak occurred in major cotton-producing regions in China, including the Yellow River and the Northwest in 2002 and 2003. In 2010, the yield loss caused by VW was 0.31 % of the total cotton yield in the U.S. [8], and losses have reached as high as 3–5 % in New Mexico [4]. The VW has not been effectively controlled mainly due to its biology characteristics, the indeterminacy of the genetic mechanism of resistance to VW and the lack of highly resistant commercial Upland cotton varieties, except for some modern Acala cotton cultivars developed in California and New Mexico [4].

Planting a resistant cultivar has long been considered the most practical, economic, and effective means of decreasing losses from VW. However, sources of VW resistance in cotton are very limited and no source of heritable immunity has been found in Upland cotton [4, 9]. Many *G. barbadense* genotypes are known to carry high levels of resistance to VW [4, 10, 11], but its resistance has not been successfully transferred into commercial Upland cotton due to hybrid breakdown except for introgressed breeding lines [4, 12, 13]. Using conventional breeding, breeders have made interspecific crosses between Upland cotton and sea-island cotton to improve the VW resistance of Upland cotton for many years, but no breakthrough has been reported because of the negative genetic correlation between lint yield and fiber quality, between lint yield and disease resistance, as well as linkage drag and hybrid breakdown [14]. This makes it challenging for breeders to realize the synchronous improvement of fiber quality, yield, and disease resistance. However, the development of molecular quantitative genetics has made it possible to locate the quantitative trait loci (QTL) for yield, fiber quality, and disease resistance, thus facilitating the use of marker-assisted selection (MAS) for genetic improvement. With the assistance of tightly linked markers to VW resistance, it is possible to transfer the resistance genes from sea-island cotton to Upland cotton.

In recent years, a great deal of progress has been made in mapping VW resistance genes and QTLs in cotton. Many genetic linkage maps have been constructed, and QTLs have been identified for VW resistance in cotton from interspecific populations of *G. hirsutum* × *G. barbadense* [2, 15–21] and from *G. hirsutum* intraspecific populations [22–27]. Some QTLs have also been detected by association mapping using a natural population, chromosome segment introgression lines and molecular mapping based on restriction-site associated DNA (RAD) sequencing technology using a RIL population [17, 28, 29]. These provided some information for further study of the QTLs/genes of VW resistance. However, most of genetic linkage maps used to identify QTLs for VW resistance in all these studies offered no more than 60 % coverage of the cotton genome. Although the map reported by Wang et al. covered 3745.9 centiMorgan (cM), an estimated 74.92 % of the tetraploid cotton genome, there were only 430 marker loci on the map [18]. Constructing a high-density linkage map is of great significance for identifying and studying the genes and QTLs of VW resistance.

To introgress the good fiber quality and VW resistance from *G. barbadense* into a commercial Upland cotton variety, we have developed the advanced backcrossing populations with the commercial Upland cotton cultivars (CCRI36) as the recipient parents, and sea-island cotton (Hai1) as the donor parent [30–32], and developed a high-density simple sequence repeat (SSR) genetic linkage map from a BC_1_F_1_ population of *G. hirsutum* × *G. barbadense*, which comprised of 2292 loci and covered 5115.16 cM of the cotton AD genome with an average marker interval of 2.23 cM [33]. To the best of our knowledge, no QTL for VW resistance were identified using any map with more than 2000 SSR loci which covered almost the whole cotton AD genome and data for VW resistance from the different the dates, generations, or environments. In this study, a whole-genome screening strategy was used to map QTLs related to VW resistance using data from different populations related to BC_1_F_1_, which is quite useful for further fine gene mapping, gene cloning, and marker assisted selection (MAS) in cotton and for further molecular design breeding.

## Results

### Differences between the parents and phenotypic variation in the populations

**Fig. 1:**
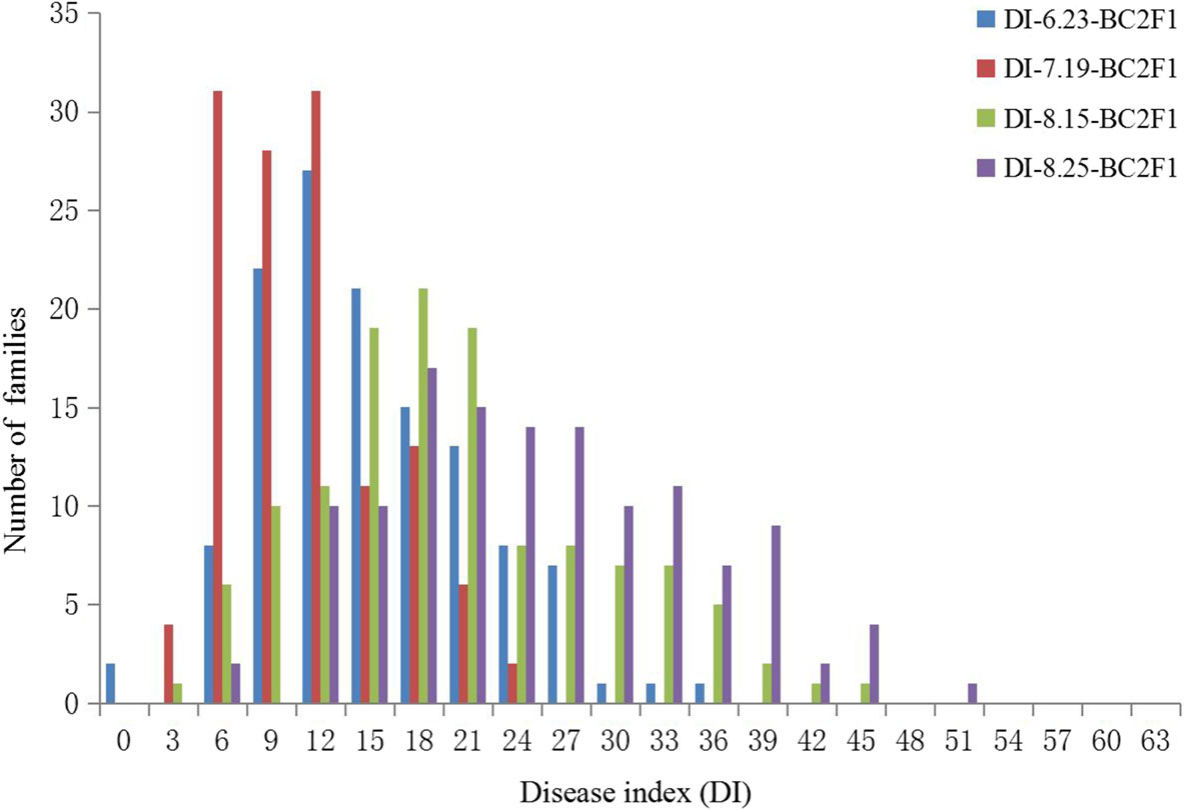
Frequency distribution of disease index (DI) in the BC_2_F_1_ population in the artificial disease nursery

**Fig. 2:**
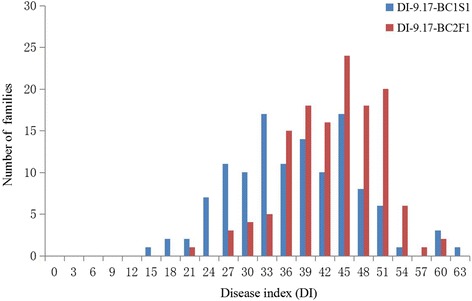
Frequency distribution of disease index (DI) in the BC_1_S_1_ and BC_2_F_1_ populations in the field

In 2005, BC_2_F_1_ family lines and their parents were evaluated for VW resistance both in the artificial disease nursery and in the field, BC_1_S_1_ family lines and their parents were also evaluated for VW resistance in the field. The recurrent parent CCRI 36 was susceptible to VW disease, and the average disease index (DI) was 30.6. Hai1 was highly resistant to VW disease with an average DI of 0.4. There was a significant difference in resistance to VW disease between the two parents. The F_1_ was also highly resistant to VW disease with an average DI of 4.3. The DI of BC_2_F_1_ family lines in the artificial disease nursery and in the field and the DI of BC_1_S_1_ family lines in field exhibited a continuous and normal distribution consistent with multi-gene inheritance for VW resistance (Figs. 1 and 2). Transgressive segregation towards to low DI value was observed (Fig. 1).

### Construction of the genetic linkage map

The high-density SSR genetic linkage map was developed from a BC_1_F_1_ population of *G. hirsutum* × *G. barbadense* [33]. Briefly, the map comprised 2292 loci and covered 5115.16 cM of the cotton AD genome with an average marker interval of 2.23 cM. The map was used to detect QTL in the BC_1_S_1_ population in the field (BC_1_S_1_-FD), BC_2_F_1_ population in the field (BC_2_F_1_-FD), and BC_2_F_1_ population in the artificial disease nursery (BC_2_F_1_-NY).

### QTL mapping in BC_2_F_1_ population in the artificial disease nursery

**Fig. 3:**
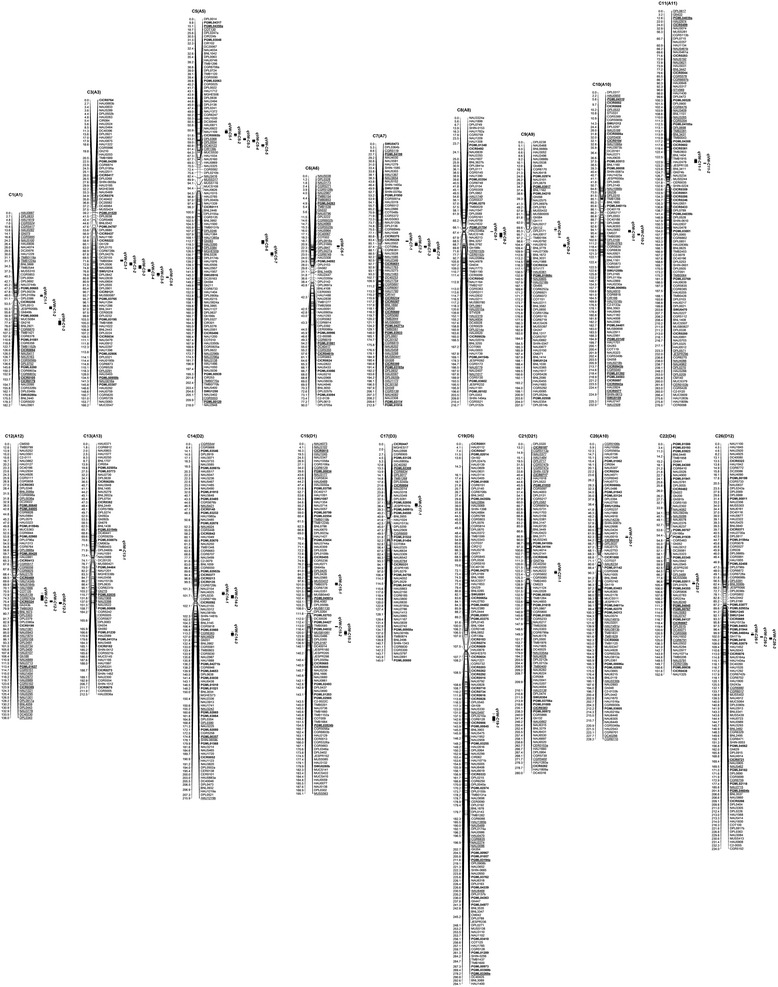
Chromosomal locations of QTLs for *Verticillium* wilt (VW) resistance in the BC_2_F_1_-FD, BC_1_S_1_-FD, and BC_2_F_1_-NY populations derived from an interspecific cross of *G. hirsutum* (CCRI36) × *G. barbadense* (Hai1)

Based on composite interval mapping, a total of 28 QTLs of VW resistance were detected in the BC_2_F_1_ population during four different dates (June 23, July 19, August 15, and August 25) in the artificial disease nursery, with 5.48–16.66 % of the total phenotypic variation explained. The 28 QTLs were located on 15 chromosomes. Chromosome (C) 5 contained 7 QTLs, and C1 and C15 contained 3 QTLs each, C10, C11, and C26 contained 2 QTLs each, and C6, C9, C13, C14, C17, C19, C20, C21, and C22 contained 1 QTL each. Of these, 19 QTLs had positive additive effects, which indicated that the *G. barbadense* alleles increased VW resistance and decreased the DI by about 2.2–7.3 (Fig. 3, Table 1).

**Table 1:** QTLs of *Verticillium* wilt (VW) resistance detected during the five dates across two generations

QTL	Gen.	Date	Env.	C	Position (cM)	Nearest Marker	Marker interval	LOD	Additive effect	PV%
qVW-C1-1	BC_2_F_1_	8.15	DN	C1	42.7	DPL0692	DPL0692-NAU3744b	3.5	5	7.88
qVW-C1-2	BC_2_F_1_	7.19	DN	C1	89.5	CGR6870	BNL2921-CGR6870	4.2	3.4	10.43
qVW-C1-3	BC_2_F_1_	7.19	DN	C1	141.6	PGML0280	NAU5163-CGR6803a	2.6	2.8	7.11
qVW-C3-1	BC_2_F_1_	9.17	FD	C3	94.2	MUSB0087	MUSB0087-PGML03765	3.3	5.7	7.32
qVW-C3-2	BC_2_F_1_	9.17	FD	C3	98	CER0028	NAU1286-DPL0232	4.3	6.5	9.57
qVW-C3-3	BC_2_F_1_	9.17	FD	C3	101.3	DPL0232	DPL0232-PGML03195	3.3	6.2	8.76
qVW-C3-4	BC_2_F_1_	9.17	FD	C3	105.3	HAU1022	TMB1898-DPL0224	5.6	7.2	11.33
qVW-C3-5	BC_2_F_1_	9.17	FD	C3	109.6	CICR0034	CICR0034-NAU2297	4.2	6.7	9.94
qVW-C3-6	BC_2_F_1_	9.17	FD	C3	113.8	HAU1396	NAU2297-DPL0321	3.9	6.1	8.16
qVW-C5-1	BC_2_F_1_	7.19	DN	C5	30.5	CIR224b	DPL0274a-PGML03048	3.3	2.7	8.1
	BC_2_F_1_	9.17	FD	C5	30.5	CIR224b		5.2	7	10.91
qVW-C5-2	BC_1_S_1_	9.17	FD	C5	32.3	CIR102	PGML03048-DPL0063	6.2	10.7	13.59
	BC_2_F_1_	9.17	FD	C5	32.3	CIR102		5.9	7.5	12.31
	BC_2_F_1_	7.19	DN	C5	32.4	CIR102		4.1	3	9.76
	BC_2_F_1_	8.15	DN	C5	32.4	CIR102		4.7	6	11.74
qVW-C5-3	BC_2_F_1_	8.15	DN	C5	38.2	HAU0746	DPL0063-DPL0724	5.5	6.5	13.52
	BC_1_S_1_	9.17	FD	C5	38.2	HAU0746		6	10.6	13.13
	BC_2_F_1_	9.17	FD	C5	38.2	HAU0746		6.5	7.9	13.46
	BC_2_F_1_	7.19	DN	C5	38.6	HAU0746		4.1	3	9.8
qVW-C5-4	BC_1_S_1_	9.17	FD	C5	39.8	PGML02063	CGR6708a-MGHES06	4.8	9.5	10.83
	BC_2_F_1_	7.19	DN	C5	39.8	PGML02063		5.1	3.2	12.01
	BC_2_F_1_	8.15	DN	C5	39.8	PGML02063		6.9	7.3	16.66
	BC_2_F_1_	9.17	FD	C5	41.2	PGML02063		4.6	6.8	10.13
qVW-C5-5	BC_2_F_1_	8.15	DN	C5	45	DPL0138	MGHES06-DPL0241	4.6	6.1	11.63
	BC_2_F_1_	9.17	FD	C5	45	DPL0138		3.6	5.8	7.72
qVW-C5-6	BC_2_F_1_	8.25	DN	C5	155.8	HAU0215	DPL0908a-NAU3824a	3.3	5.8	9.22
qVW-C5-7	BC_2_F_1_	8.25	DN	C5	168.6	CGR5925a	COT010-NAU2296b	2.9	5.1	7.09
qVW-C6-1	BC_2_F_1_	8.15	DN	C6	0.5	NAU5038	NAU5038-CGR5128b	2.9	−4.9	7.13
qVW-C7-1	BC_1_S_1_	9.17	FD	C7	75.3	CGR6894b	DPL0136-NAU1048	2.8	7.2	6.05
qVW-C7-2	BC_1_S_1_	9.17	FD	C7	85	NAU2002	NAU1048-COT096a	3.5	7.9	7.38
qVW-C7-3	BC_1_S_1_	9.17	FD	C7	88.1	CGR6381	COT096a-NAU1763	3.4	7.8	7.08
qVW-C7-4	BC_1_S_1_	9.17	FD	C7	100.4	DC40253	NAU1483-DC40253	4	9.2	10.09
qVW-C8-1	BC_2_F_1_	9.17	FD	C8	67.4	HAU3346a	PGML01704-BNL3257	3	5.1	5.76
qVW-C8-2	BC_2_F_1_	9.17	FD	C8	79.7	CER0152c	CER0152c-NAU2086a	3.3	6.7	8.43
qVW-C9-1	BC_2_F_1_	6.23	DN	C9	61.1	MUSB0009	DPL0783-Gh112	2.7	−3.5	6.86
qVW-C9-2	BC_2_F_1_	9.17	FD	C9	117.1	STV177	HAU2730b-HAU0361	3.6	5.7	7.07
qVW-C10-1	BC_2_F_1_	6.23	DN	C10	150.7	NAU2869	NAU2869-HAU0230a	2.8	3.8	8.09
qVW-C10-2	BC_2_F_1_	6.23	DN	C10	161	CGR6818	NAU4910-CIR166	2.9	3.2	5.77
qVW-C11-1	BC_2_F_1_	8.15	DN	C11	63.9	NAU5461b	MUSS281-NAU5621a	5.5	6.7	13.83
qVW-C11-2	BC_2_F_1_	8.15	DN	C11	70.6	NAU5461a	NAU5621a-CICR0283	4.7	6.1	11.54
qVW-C12-1	BC_1_S_1_	9.17	FD	C12	103.5	HAU0734	DPL01491-DPL0400	2.7	9.3	7.53
qVW-C12-2	BC_1_S_1_	9.17	FD	C12	107.8	HAU0107	DPL0400-Gh188	3.7	8.9	7.75
qVW-C12-3	BC_1_S_1_	9.17	FD	C12	109.9	NAU4889	Gh188-NAU3713	3.6	10.2	8.94
qVW-C13-1	BC_2_F_1_	6.23	DN	C13	47.3	CICR0382	DPL0754-BNL2449	3.4	3.4	6.62
qVW-C14-1	BC_2_F_1_	9.17	FD	C14	81.6	C2-0079	NAU3499-CGR6550	4.4	6.4	8.7
qVW-C14-2	BC_2_F_1_	9.17	FD	C14	88.9	PGML02953b	CGR6550-CICR0377	2.9	5.5	6.43
qVW-C14-3	BC_2_F_1_	6.23	DN	C14	184.6	NAU5465	SHIN-0659b-CICR0052	2.6	3.2	5.71
qVW-C15-1	BC_2_F_1_	8.25	DN	C15	43.9	CGR5834	NAU3177-HAU3050a	3.8	−5.8	9.5
qVW-C15-2	BC_1_S_1_	9.17	FD	C15	141.9	NAU0861	NAU3680-DPL0437	2.6	−7	5.87
qVW-C15-3	BC_1_S_1_	9.17	FD	C15	152	TMB1664	COT059-PGML02824a	2.9	−7.1	6.05
	BC_2_F_1_	8.15	DN	C15	155.5	PGML02824b	TMB1664-CGR6308b	2.9	−4.8	6.92
qVW-C15-4	BC_2_F_1_	7.19	DN	C15	161.2	CGR5056a	PGML02824a-CER0013	3.4	−2.9	9.02
qVW-C17-1	BC_2_F_1_	7.19	DN	C17	5.3	HAU0800a	CGR6905-DC40292	2.6	2.2	5.48
qVW-C19-1	BC_2_F_1_	6.23	DN	C19	185.6	HAU1385b	DPL0192-NAU5489	4.5	4.3	10.16
qVW-C20-1	BC_2_F_1_	8.25	DN	C20	22.2	CICR0254	CIR094-NAU4071	3.6	−5.6	9.01
qVW-C21-1	BC_2_F_1_	7.19	DN	C21	111.3	Gh074a	CICR0046-DC40250	4.2	3.1	9.99
qVW-C22-1	BC_2_F_1_	8.25	DN	C22	149.7	CER0139b	TMB0206-CICR0438	2.8	−5	6.93
qVW-C26-1	BC_2_F_1_	8.15	DN	C26	164.5	DPL0915	PGML04562-DPL0915	3.9	−5.9	11.12
qVW-C26-2	BC_2_F_1_	8.15	DN	C26	169.4	NAU3905	NAU4914-PGML04182	3.5	−5	7.95
qVW-C26-3	BC_1_S_1_	9.17	FD	C26	172.4	CGR6759	DPL0890-PGML02118	3	−7.4	6.48

Note: 8.15 means that the DI was investigated at August 15; *DN* the artificial disease nursery, *FD* the field, *Gen* generation, *Env* environment, *C* chromosome, *PV%* phenotypic variation explained; positive additive effect indicates that the locus derived from Hai1 decreased the value of DI

#### QTLs of VW resistance for June 23

Six QTLs, qVW-9-1, qVW-10-1, qVW-10-2, qVW-13-1, qVW-14-3 and qVW-19-1, were identified in the BC_2_F_1_ population in the artificial disease nursery and found on 5 chromosomes, each explaining 5.71–10.16 % of the total phenotypic variation. All QTLs except qVW-9-1 had positive additive effects, which indicated that the Hai1 alleles increased VW resistance and decreased VW DI by 3.2–4.3.

#### QTLs of VW resistance for July 19

Nine QTLs, qVW-1-2, qVW-1-3, qVW-5-1, qVW-5-2, qVW-5-3, qVW-5-4, qVW-15-4, qVW-17-1 and qVW-21-1, were detected in the BC_2_F_1_ population in the artificial disease nursery, each explaining 5.48–12.01 % of the total phenotypic variation. These were located on 5 chromosomes, four QTLs were found on Chr5 alone. All QTLs except qVW-15-4 had positive additive effects, which indicated that the Hai1 alleles increased VW resistance and decreased VW DI by 2.2–3.4.

#### QTLs of VW resistance for August 15

Eleven QTLs, qVW-1-1, qVW-5-2, qVW-5-3, qVW-5-4, qVW-5-5, qVW-6-1, qVW-11-1, qVW-11-2, qVW-15-3, qVW-26-1 and qVW-26-2, were identified in the BC_2_F_1_ population in the artificial disease nursery, each explaining 6.92–16.66 % of the total phenotypic variation. The QTLs were located on 6 chromosomes, four on Chr5. All QTLs except qVW-6-1, qVW-15-3, qVW-26-1 and qVW-26-2 had positive additive effects, which indicated that the Hai1 alleles increased VW resistance and decreased VW DI by 5.0–7.3. In addition, qVW-C26-1 and qVW-C26-2 were both located on C26 and explained 11.12 and 7.95 % of the phenotypic variation, respectively. The CCRI36 alleles increased VW resistance and decreased VW DI by 5.0 and 5.9, respectively.

#### QTLs of VW resistance for August 25

Five QTLs, including qVW-5-6, qVW-5-7, qVW-15-1, qVW-20-1 and qVW-22-1, were detected in the BC_2_F_1_ population in the artificial disease nursery and located on 4 chromosomes, each explaining 6.93–9.50 % of the total phenotypic variation. Two QTLs (qVW-5-6 and qVW-5-7) had positive additive effects, and they were located on the same chromosome, C5. They explained 9.22 and 7.09 % of the phenotypic variation, respectively. The Hai1 alleles increased VW resistance and decreased VW DI by 5.8 and 5.1, respectively.

qVW-C5-2, qVW-C5-3 and qVW-C5-4 on C5 were detected in the two dates on July 19 and August 15, and were localized in the marker intervals PGML03048-DPL0063, DPL0063-DPL0724, and CGR6708a-MGHES06, respectively. qVW-C5-2 explained 9.76–11.74 % of the phenotypic variation, and the *G. barbadense* allele decreased the DI by about 3.0–6.0. qVW-C5-3 explained 9.8–13.52 % of the phenotypic variation, and the *G. barbadense* allele decreased the DI by about 3.0–6.5. qVW-C5-4 explained 12.01–16.66 % of the phenotypic variation, and the *G. barbadense* allele decreased the DI by about 3.2–7.3.

### QTL mapping in BC_2_F_1_ and BC_1_S_1_ populations in the field

Based on composite interval mapping, a total of 13 QTLs associated with VW resistance, qVW-5-2, qVW-5-3*,* qVW-5-4, qVW-7-1, qVW-7-2, qVW-7-3, qVW-7-4, qVW-12-1, qVW-12-2, qVW-12-3, qVW-15-2, qVW-15-3 and qVW-26-3, were detected in the BC_1_S_1_ population on September 17, explaining 5.87–13.59 % of the total phenotypic variation. These 13 QTLs were located on C5, C7, C12, C15 and C26. C7 contained 4 QTLs, C5 and C12 contained 3 QTLs each, C15 contained 2 QTLs and C26 contained 1 QTL. All QTLs except qVW–15-2, qVW-15-3, and qVW-26-3 had positive additive effects, which indicated that the *G. barbadense* allele decreased the DI by about 7.2–10.7 (Fig. 3, Table 1).

A total of 16 QTLs for VW resistance, qVW-3-1, qVW-3-2, qVW-3-3, qVW-3-4, qVW-3-5, qVW-3-6, qVW-5-1, qVW-5-2, qVW-5-3, qVW-5-4, qVW-5-5, qVW-8-1, qVW-8-2, qVW-9-2, qVW-14-1 and qVW-14-2, were detected in the BC_2_F_1_ population on September 17, explaining 5.76–13.46 % of the total phenotypic variation. The 16 QTLs were located on C3, C5, C8, C9 and C14. C3 contained 6 QTLs, C5 contained 5 QTLs, C8 and C14 each contained 2 QTLs and C9 contained 1. All 16 QTLs had positive additive effects, which indicated that the *G. barbadense* allele decreased the DI by about 5.1–7.9 (Fig. 3, Table 1).

### Stability of QTLs over multiple generations, environments and dates

A total of 48 QTLs of VW resistance were detected in the BC_2_F_1_ and BC_1_S_1_ populations and the five dates. These were located on 19 chromosomes. C5 contained 7 QTLs, C3 contained 6 QTLs, C7 and C15 each contained 4 QTLs, C1, C12, C14 and C26 each contained 3 QTLs, C8, C9, C10 and C11 each contained 2 QTLs, and C6, C13, C17, C19, C20, C21 and C22 each contained 1 QTL. Of these, 37 QTLs (77.08 %) had positive additive effects, which indicated that the *G. barbadense* allele decreased the DI (Fig. 3, Table 1).

**Fig. 4:**
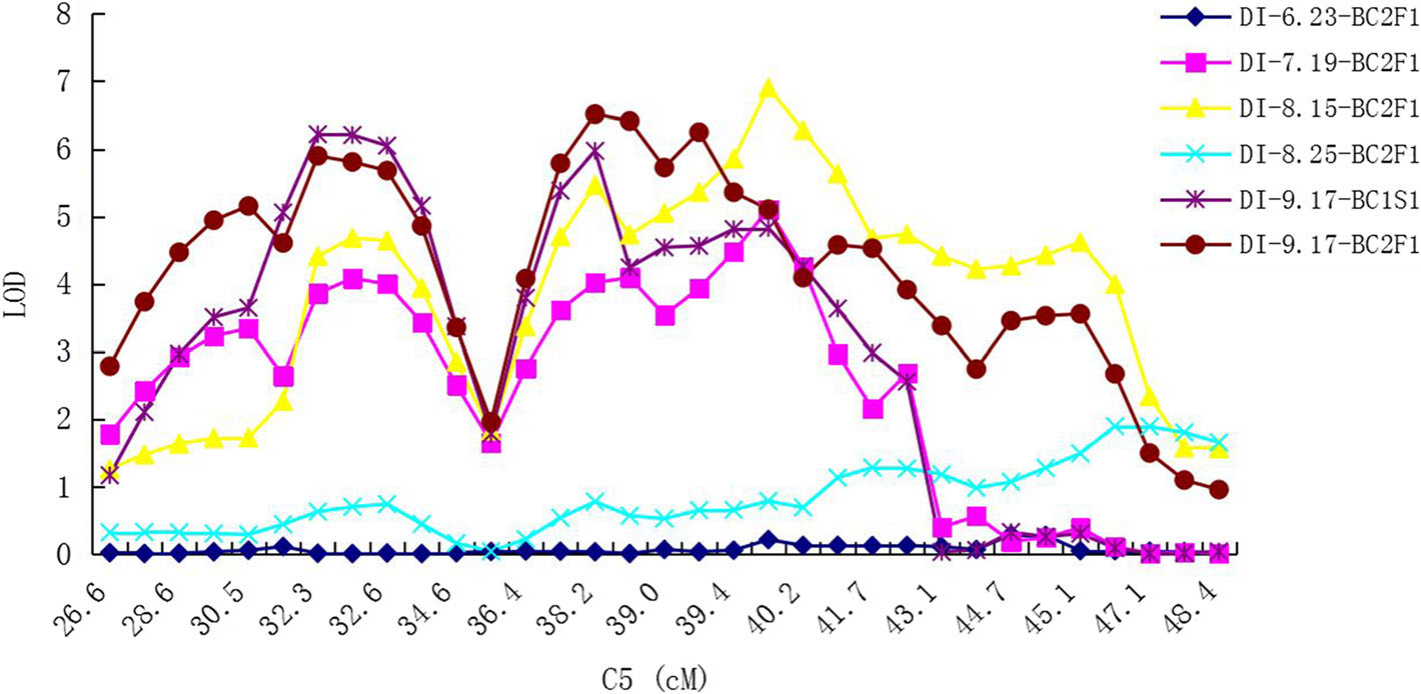
Graphic display of QTLs related to VW resistance within the 26.6 cM–48.4 cM interval on C5 using WinQTLCart 2.0 based on the interspecific backcross populations of CCRI36 × Hai1

Of these 48 QTLs, 3 QTLs (qVW-C5-2, qVW-C5-3, qVW-C5-4) were detected in two generations (BC_2_F_1_, BC_1_S_1_), in two environments (the field, the artificial disease nursery) and in the three dates (July 19, August 15 and September 17), explaining 9.76–16.66 % of the phenotypic variation. The Hai1 allele decreased the DI by approximately 3.0–10.7, increasing VW disease resistance. One QTL (qVW-C5-1) was simultaneously detected in both environments in the artificial disease nursery and in the field for BC_2_F_1_, explaining 8.10–10.91 % of the phenotypic variation, and the Hai1 allele decreased the DI by approximately 2.7–7.0. One QTL (qVW-C5-5) was also simultaneously detected in two environments or dates on August 15 in the artificial disease nursery and on September 17 in the field for BC_2_F_1_, explaining 7.72–11.63 % of the phenotypic variation. The Hai1 allele decreased the DI by approximately 5.8–6.1 (Figs. 3 and 4, Table 1). One QTL (qVW-C15-3) was simultaneously detected on September 17 in BC_1_S_1_ in the field and in BC_2_F_1_ on August 15 in the artificial disease nursery, explaining 6.05–6.92 % of the phenotypic variation. And the CCRI36 allele decreased the DI by about 4.8–7.1. A total of six QTLs were stably detected in different generations, environments, and/or different dates. These stable QTLs could be used for MAS breeding.

### Meta-analysis of QTLs

**Fig. 5:**
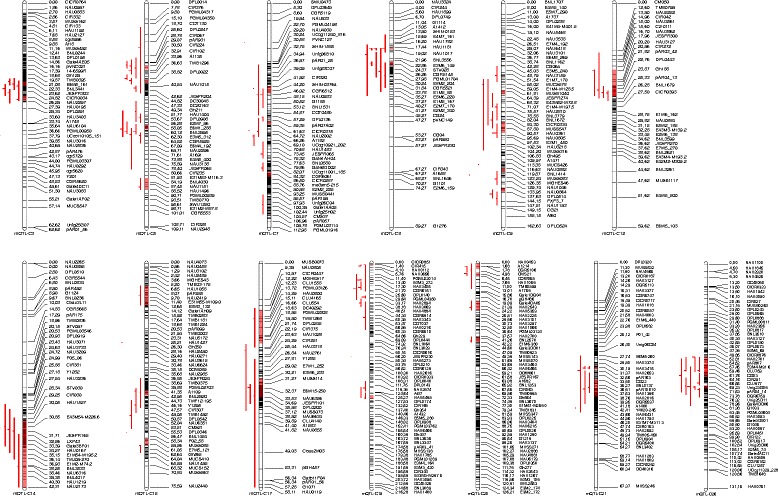
QTLs and QTL hotspots for *Verticillium* wilt (VW) resistance on the consensus map by a meta-analysis

In the Meta-analysis, a total of 17 QTL hotspot regions were identified on 13 chromosomes of the consensus map, including C3, C5, C7, C8, C9, C12, C14, C15, C17, C19, C20, C21 and C26 (Fig. 5, Table 2). 7 QTL hotspot regions (c5-VW-Hotspot-2, c7-VW-Hotspot-1, c7-VW-Hotspot-2, c8-VW-Hotspot-1, c19-VW-Hotspot-1, c21-VW-Hotspot-1 and c26-VW-Hotspot-1) were consistent with those identified previously by Zhang et al. and Said et al. [13, 34] (Table 2), and the other ten were new, unreported hotspot regions. Two QTL hotspot regions were on C5, C7, C9 and C19, respectively. Of 17 QTL hotspot regions, c3-VW-Hotspot-1, c5-VW-Hotspot-1, c8-VW-Hotspot-1 and c26-VW-Hotspot-1 had more QTLs.

**Table 2:** QTL hotspots identified for *Verticillium* wilt (VW) resistance on the consensus map by meta-analysis

Hotspot name	C	Position (cM)	No. of QTLs	No. of QTLs in this paper	Reported previously
c3-VW-Hotspot-1	C3	16–28 cM	7	6	
c5-VW-Hotspot-1	C5	28–43 cM	7	5	
c5-VW-Hotspot-2	C5	47–68 cM	5	2	Zhang et al. 2015 [13] and Said et al. 2015 [34]
c7-VW-Hotspot-1	C7	43–59 cM	4	1	Zhang et al. 2015 [13]
c7-VW-Hotspot-2	C7	64–85 cM	5	3	Zhang et al. 2015 [13]
c8-VW-Hotspot-1	C8	17–34 cM	8	2	Zhang et al. 2015 [13]
c9-VW-Hotspot-1	C9	55–85 cM	6	1	
c9-VW-Hotspot-2	C9	124–147 cM	5	0	
c12-VW-Hotspot-1	C12	17–26 cM	4	3	
c14-VW-Hotspot-1	C14	26–43 cM	5	3	
c15-VW-Hotspot-1	C15	42–63 cM	6	3	
c17-VW-Hotspot-1	C17	11–36 cM	4	1	
c19-VW-Hotspot-1	C19	1–26 cM	5	0	Zhang et al. 2015 [13]
c19-VW-Hotspot-2	C19	120–132 cM	4	1	
c20-VW-Hotspot-1	C20	1–26 cM	4	1	
c21-VW-Hotspot-1	C21	30–44 cM	6	1	Zhang et al. 2015 [13]
c26-VW-Hotspot-1	C26	54–79 cM	9	3	Zhang et al. 2015 [13]

36 QTLs from this paper were distributed in 15 hotspot regions, 29 of them were in 12 hotspot regions and were all from *G. barbadense* (Table 3). It showed that QTL-rich regions on the map of CCRI36 × Hai1 BC_1_F_1_ were consistent with the QTL hotspot regions (Table 3, Additional file 1). These consensus QTL regions (QTL-rich regions and QTL hotspot regions) could be worthy of further research and used for MAS.

**Table 3:** QTLs identified using interspecific backcross populations on the map of CCRI36 × Hai1 BC_1_F_1_ and the corresponding hotspots

On the map of CCRI36 × Hai1 BC_1_F_1_			On the consensus map
QTL-rich regions	QTL	Direction	Hotspot name and range
	qVW-C1-1	Hai1	
	qVW-C1-2	Hai1	
	qVW-C1-3	Hai1	
C3-VW-QTL-rich-1: 93–114 cM	qVW-C3-1	Hai1	c3-VW-Hotspot-1: 16–28 cM
	qVW-C3-2	Hai1	
	qVW-C3-3	Hai1	
	qVW-C3-4	Hai1	
	qVW-C3-5	Hai1	
	qVW-C3-6	Hai1	
C5-VW-QTL-rich-1: 27–47 cM	qVW-C5-1	Hai1	c5-VW-Hotspot-1: 28–43 cM
	qVW-C5-2	Hai1	
	qVW-C5-3	Hai1	
	qVW-C5-4	Hai1	
	qVW-C5-5	Hai1	
	qVW-C5-6	Hai1	c5-VW-Hotspot-2: 47–68 cM
	qVW-C5-7	Hai1	
	qVW-C6-1	CCRI36	
C7-VW-QTL-rich-1: 75–100 cM	qVW-C7-1	Hai1	c7-VW-Hotspot-1: 43–59 cM
	qVW-C7-2	Hai1	c7-VW-Hotspot-2: 64–85 cM
	qVW-C7-3	Hai1	
	qVW-C7-4	Hai1	
	qVW-C8-1	Hai1	c8-VW-Hotspot-1: 17–34 cM
	qVW-C8-2	Hai1	
	qVW-C9-1	CCRI36	
	qVW-C9-2	Hai1	c9-VW-Hotspot-1: 55–85 cM
	qVW-C10-1	Hai1	
	qVW-C10-2	Hai1	
	qVW-C11-1	Hai1	
	qVW-C11-2	Hai1	
C12-VW-QTL-rich-1: 93–112 cM	qVW-C12-1	Hai1	c12-VW-Hotspot-2: 17–26 cM
	qVW-C12-2	Hai1	
	qVW-C12-3	Hai1	
	qVW-C13-1	Hai1	
	qVW-C14-1	Hai1	c14-VW-Hotspot-1: 26–43 cM
	qVW-C14-2	Hai1	
	qVW-C14-3	Hai1	
	qVW-C15-1	CCRI36	
C15-VW-QTL-rich-1: 141–165 cM	qVW-C15-2	CCRI36	c15-VW-Hotspot-1: 42–63 cM
	qVW-C15-3	CCRI36	
	qVW-C15-4	CCRI36	
	qVW-C17-1	Hai1	c17-VW-Hotspot-1: 11–36 cM
	qVW-C19-1	Hai1	c19-VW-Hotspot-2: 120–132 cM
	qVW-C20-1	CCRI36	c20-VW-Hotspot-1: 1–26 cM
	qVW-C21-1	Hai1	c21-VW-Hotspot-1: 30–44 cM
	qVW-C22-1	CCRI36	
C26-VW-QTL-rich-1: 158–177 cM	qVW-C26-1	CCRI36	c26-VW-Hotspot-1: 54–79 cM
	qVW-C26-2	CCRI36	
	qVW-C26-3	CCRI36	

## Discussion

### Map used to identify QTLs for *Verticillium* wilt (VW) resistance

To identify QTL associated with VW resistance from *G. barbadense*, some genetic maps were developed from crosses between *G. hirsutum* and *G. barbadense* or *G. hirsutum* and an introgressed line. For example, Zhang et al. detected QTLs for VW resistance based on a 392 SSR polymorphic loci linkage map covering 2895 cM or about 57.90 % of the cotton genome using the BIL population of SG 747 × Giza 75 [13]. Fang et al. constructed a 292-marker genetic linkage map for the BIL population of SG 747 × Pima S-7 [15]. It covered 1226 cM or about 27 % of the cotton genome. Yang et al. constructed two genetic linkage maps [19]. One of these included 35 linkage groups with an average distance between two markers of 8.7 cM with 219 SSR loci. They covered 1772.5 cM or approximately 31.93 % of the tetraploid cotton genome from BC_1_ of Hai 7124 × Junmian 1. Another genetic linkage map with 420 genome-wide loci using F_2_ of Hai 7124 × Junmian 1, with an average distance of 7.3 cM per marker covered 2726.9 cM or approximately 49.10 % of the tetraploid cotton genome. Wang et al. constructed a genetic linkage map with 430 marker loci using F_2_ population of XinLuZao1 × Hai7124, with an average distance of 8.71 cM per marker and covered 3745.9 cM, which is an estimated 74.92 % of the total recombination length of the tetraploid cotton genome [18]. Bolek et al. constructed eleven linkage groups using an F_2_ population derived from the interspecific cross of Pima S-7 and Acala 44, consisting of 35 markers and spanning 531 cM (approximately 10.62 % of the tetraploid cotton genome) with an average distance of 15.17 cM [2]. Gao et al. constructed a genetic linkage map with 99 marker loci using an F_2_ population of Handan208 × Pima90 [20]. It had an average distance of 18.61 cM per marker and covered 1842.8 cM or approximately 36.8 % of the tetraploid cotton genome. Fang et al. constructed a genetic linkage map with 882 marker loci using the RIL population of TM-1 × NM24016 [16]. It had an average distance of 7.06 cM per marker and covered 2267 cM, which is an estimated 55.7 % of the total recombination length of the tetraploid cotton genome. NM24016 is an inbred Upland cotton line with considerable but stable introgression from several *G. barbadense* lines. Ning et al. constructed a genetic linkage map with 279 marker loci using a RIL population of Prema × 86–1 in which Prema was an introgressed line from *G. thurberi* and *G. barbadense* [23]. This map covered 1576.25 cM, which is an estimated 35.42 % of the total recombination length of the tetraploid cotton genome. Using the same population, Wang et al. constructed a molecular map based on restriction-site associated DNA (RAD) sequencing technology, covering 3499.69 cM, which is an estimated 69.99 % of the total recombination length of the tetraploid cotton genome [29].

In addition, some genetic maps have also been developed using intraspecific populations of *G. hirsutum* to identify QTL for VW resistance. Zhang et al. constructed a genetic linkage map with 141 marker loci using an F_2_ population of LHB22 × JM11 [22]. It had an average distance of 8.11 cM per marker and covered 1143.1 cM, which is an estimated 22.86 % of the tetraploid cotton genome. Jiang et al. constructed a genetic linkage map using F_2_ segregating population of 60182 × Junmian 1, with 139 loci, covering 1165 cM [24]. It had an average distance of 8.38 cM between two markers, or 25.89 % of the length of the cotton genome. Ge et al. constructed a genetic linkage map with 122 marker loci using an F_2_ population of Chang96 × Junmian 1 [25]. It had 33 linkages and covered 1172.7 cM or approximately 23.5 % of the tetraploid cotton genome. Wang et al. constructed a genetic linkage map with 10 marker loci using F_2_ population of Lumianyan22 × Luyuan343 [26]. It had three linkages and covered 108.9 cM. Yang et al. constructed a genetic linkage map with 115 marker loci using an RIL population of 5026 × Li 8 [27]. It had 20 linkage groups and covered 560.1 cM or approximately 11.2 % of the tetraploid cotton genome.

Only 1–18 QTLs for VW resistance were detected using these maps in previous reports. In the previous reports, most of these linkage maps used to identify QTLs associated with VW resistance had no more than 2000 SSR markers and covered less than 60 % of the cotton genome [2, 15, 16, 19, 22–24]. It is difficult to identify more QTLs for VW resistance from *G. barbadense* using the limited number of markers and low coverage of the whole cotton genome.

In this study, the genetic map comprised 2292 SSR marker loci and covered 5115.16 cM of the cotton genome [33]. A total of 48 QTLs for VW resistance were detected and distributed on 19 chromosomes. This showed that more QTLs for VW were found and located in more chromosomes and were mapped to a narrower interval with tightly linked markers using our map than maps of the traits reported in previous papers.

### Distribution of QTLs through the whole genome

QTL clusters of different fiber quality and disease resistance have also been observed in previous studies [1, 35–41]. The current results showed some QTL-rich regions on C3, C5, C7, C12, C15 and C26 for VW resistance. Six QTLs were detected within a C3 region (93–114 cM) between MUSB0087 and DPL0321. A C5 region (27–47 cM) between DPL0274a and DPL0241 harbored 5 QTLs and a C7 region (75–100 cM) between DPL0136 and DC40253 carried 4 QTLs. Three QTLs each were found within a specific range on C12 (93–112 cM) between DPL01491 and NAU3713, within a specific range on C15 (141–165 cM) between NAU3680 and CER0013, and within a specific range on C26 (158–177 cM) between PGML04562 and PGML02118 (Table 1, 3, Fig. 3). Importantly, the 5 QTLs for VW resistance that clustered on C5 were stably detected in different generations, different environments and/or different dates.

In this paper, a total of 48 QTLs for VW resistance were detected in BC_1_S_1_ and BC_2_F_1_ populations using the high-density linkage map and they were distributed on 19 chromosomes. Here, 33 (68.75 %) of them were located in the A subgenome, involving 11 chromosomes (C1, C3, C5, C6, C7, C8, C9, C10, C11, C12 and C13), and 15 QTLs were located on the D subgenome, involving 8 chromosomes (C14, C15, C17, C19, C20, C21, C22 and C26). This indicated that the A subgenome harbored more QTLs or genes for VW resistance than the D subgenome. This was consistent with results reported by Yang et al. [19], Ning et al. [23], and Bolek et al. [2]. However, they are not consistent with the idea that the D subgenome makes a greater contribution to genetic control of resistance traits than the A subgenome does [42, 43]. This could be relevant to the conclusion that more QTLs cluster on the A subgenome in this study. It may be because different materials were used in different studies.

### QTLs for *Verticillium* wilt (VW) resistance from *G. barbadense*

In this paper, a total of 48 QTLs for VW resistance were detected in two generations (BC_2_F_1_, BC_1_S_1_) using a high-density linkage map. Of these, 37 (77.08 %) QTLs had positive additive effects, which indicated that the *G. barbadense* allele increased VW resistance and decreased the DI by about 2.2–10.7. Six QTLs were stably detected in two different generations and different environments and dates. Of these, 5 QTLs clustered on C5 and 1 was located on C15.

The six QTLs detected were found to be on the same chromosomes or subgenomes reported in previous studies, and they had shared SSR markers [17, 19, 21–23]. qVW-C7-1 located on C7 for VW resistance was the same as Yang’s qVL-A7-1 F_2_ [19], based on shared markers of NAU1048. qVW-C19-1 may be the same as qVL-D5-1BC_1_S_2_592 in Yang et al. [19]. These were linked to the common marker BNL1878. qVW-C5-2 was same as QTL (*qVW-A5-1*) reported by Ning et al. with the linked common marker DC20067 [23]. The qVW-C21-1 here detected for VW resistance may be the same as the qVW-c21-1 reported in Zhang et al. with the linked common markers HAU0423 and CGR5602 [22]. qVW-C9-2 was assigned to the same chromosome region as the qFD1711-11-4.01 reported by Wu et al. with the linked common marker BNL3031 [21]. qVW-C3-5 may be the same as qRV991-A3-1 in Wang et al. [17]. These were linked to the common marker NAU3479. We could not correlate the other QTLs for VW resistance to those found in other studies due to the lack of common markers, although some QTLs were on the same chromosomes [15, 18, 20, 22, 23, 26, 27, 29, 36]. The more frequently informative molecular markers are used in the cotton community, the greater the likelihood that QTL conditioning VW resistance among various cotton germplasm lines could be tagged. In this way, the other 42 QTLs for VW resistance could be considered newly identified by this map.

In summary, 6 stable QTLs (qVW-C5-1, qVW-C5-2, qVW-C5-3, qVW-C5-4, qVW-C5-5, and qVW-C15-3) were detected in two or more populations or dates in this map and 6 consensus QTLs (qVW-C7-1, qVW-C19-1, qVW-C5-2, qVW-C21-1, qVW-C9-2, qVW-C3-5) were also reported in the previous studies. These 11 stable or consensus QTLs (qVW-C5-1, qVW-C5-2, qVW-C5-3, qVW-C5-4, qVW-C5-5, qVW-C15-3, qVW-C7-1, qVW-C19-1, qVW-C21-1, qVW-C9-2, qVW-C3-5) could be used for MAS. Among 48 QTLs, there were 42 new unreported QTLs, of which 31 QTLs were from *G. barbadense*.

By meta-analysis of QTLs, 17 QTL hotspot regions were identified on 13 chromosomes of the consensus map (Fig. 5, Table 2). Of them, 7 QTL hotspot regions were consistent with those identified previously by Zhang et al. and Said et al. [13, 34] (Table 2) and another 10 were new, unreported hotspot regions. 36 (75 %) QTLs from this paper were distributed in 15 hotspot regions, of which 29 QTLs were in 12 hotspot regions and were all from *G. barbadense* (Table 3). All QTL-rich regions on the map of CCRI36 × Hai1 BC_1_F_1_ were consistent with the QTL hotspot regions of the consensus map (Table 3, Additional file 1). These consensus QTL regions (QTL-rich regions or QTL hotspot regions) could be worthy of further research and used for MAS.

### Further application of QTLs for *Verticillium* wilt (VW) resistance

Chromosome segment substitution lines (CSSLs) with the two commercial Upland cotton cultivars (CCRI36 and CCRI45) were developed as the recipient parents to introgress genes and QTLs associated with good fiber quality and VW resistance from *G. barbadense* into a commercial Upland cotton variety, *G. barbadense* (Hai1) served as a donor parent. The fiber yield and quality traits of these CSSLs have been evaluated in previous works [30–32, 44–46]. VW resistance in these CSSLs is being evaluated. The minor difference between CSSLs and the recurrent parent lies in the donor chromosome segment from *G. barbadense* in *G. hirsutum* genetic background, reducing the interference of the genetic background. In this way, CSSLs are an ideal material for genetic studies on quantitative traits. The present study provides molecular information for fine gene mapping, gene cloning, gene pyramiding, and marker-assisted breeding for improving VW resistance and lays a solid foundation for further molecular design breeding throughout the cotton genome.

## Conclusions

In the present study, a high-density SSR genetic linkage map covering the whole cotton genome and data associated with VW resistance from different dates in populations of different generations in the field and in the artificial disease nursery were used to identify QTLs associated with VW resistance and never-before-reported QTLs were detected. Here, 48 VW resistance QTLs were mapped based on a 5115.16 cM linkage map with 2292 SSR marker loci. This work provides useful information for further comparative analysis and improving understanding of the genetic basis of VW resistance in cotton. Here, six QTLs were found to be stable, of which 5 QTLs clustered on C5 and 1 on C15. In this paper, six QTLs were the same or similar to previously reported QTLs and 42 QTLs were new. Of these 42, 31 QTLs were from *G. barbadense*. 17 QTL hotspot regions for VW resistance were identified and 10 of them were new, unreported hotspot regions. 29 QTLs in 12 hotspot regions were all from G. barbadense. These stable and consensus QTLs provide important molecular information for MAS in Upland cotton breeding aimed at improving the resistance to VW and may lay a better foundation for gene cloning and further study.

## Methods

### Plant materials

The mapping population with 135 BC_1_F_1_ plants was derived from an interspecific cross of *G. hirsutum* (CCRI36) × *G. barbadense* (Hai1), in which CCRI36 is a susceptible cultivar (*G. hirsutum* L.) and Hai1 is a highly resistant line (*G. barbadense* L.) with super fiber quality and the dominant glandless gene [33, 47].

The CCRI36× Hai1 F_1_ was made in Henan Province during the summer of 2003 and F_1_ plants, which served as the male parent, were backcrossed with the recurrent parent CCRI36 in Hainan Province. BC_1_F_1_ seeds were harvested during the spring of 2003. In 2004, the glanded plants in the BC_1_F_1_ population were pulled out at the seedling stage, and BC_1_F_1_ plants, which served as the male parent, were backcrossed with CCRI36, and the hybrid seeds originating from the same BC_1_F_1_ plants were harvested together (BC_2_F_1_ seeds were obtained). BC_1_F_1_ plants were self-pollinated and BC_1_S_1_ seeds were harvested.

### Field evaluation of *Verticillium* wilt (VW) disease resistance for BC_2_F_1_ and BC_1_S_1_ populations

A field in the Anyang experiment farm at the Institute of Cotton Research of Chinese Academy of Agricultural Sciences, Anyang, Henan Province, China, had been consecutively planted for more than 30 years with cotton. So it had a relatively high and uniform density of *V. dahliae* with both of the defoliating and nondefoliating populations and could be used as a natural nursery to screen for VW resistance breeding lines. In April 2005, 133 BC_2_F_1_ and 121 BC_1_S_1_ families (BC_2_F_1_-FD and BC_1_S_1_-FD) and the two parental lines (CCRI36 and Hai1) and its F_1_ were planted in this field in single-row plots at one replication for per every family and in two-row plots for the two parents and F_1_. Each row was 8 m long and 0.8 m wide with 32 plants. The glanded plants in BC_2_F_1_ and BC_1_S_1_ were pulled out at the seedling stage.

### Preparation of inoculum and the artificial disease nursery evaluation of *Verticillium* wilt (VW) for BC_2_F_1_ population

A defoliant, moderate pathogenic Anyang strain of *Verticillium dahliae*, was a dominant pathogenic strain, isolated from the seriously infected field of cotton at Anyang, Henan Province, China. Before sowing cotton, the Anyang strain was cultured with cotton seeds with fuzz at 25 °C for 10 days. And the dry culture was grinded and placed uniformly in the artificial disease nursery, with the inoculum concentration of 45 g of dry culture per 1 square meter of the disease nursery with conidial content of 1 × 10^8^ per 1 g of the dry culture. This inoculation procedure yielded a mean DI of ca. 50 on the susceptible check variety in the normal conditions. The VW resistance for the 133 BC_2_F_1_ families and their parents were evaluated on June 23, July 19, August 15, and August 25 in 2005 in the artificial disease nursery.

The artificial disease nursery consists of a series of cement pools used for the artificial inoculation. Every pool is 20 m long and 2.5 m wide. The test was arranged in a randomized complete block design (RCBD) with 3 replicates and one row (plot) for every family. Each row was 2.5 m long and 0.6 m apart with 15 plants.

### Phenotyping

The leaf tissue damage in the seedling and the maturity stages were classified into five grades which is a national standard in *Verticillium* resistant scoring in China [19, 48]. The grades scored as 1 and 2 were considered as resistant, and grades 3 and 4 as susceptible to VW.

- <25 % chlorotic/necrotic leaves, grades 1
- 25–50 % chlorotic/necrotic leaves, grades 2
- 50–75 % chlorotic/necrotic leaves, grades 3
- >75 % chlorotic/necrotic leaves, grades 4

The disease grade of each plant in BC_2_F_1_ was recorded by the leaf symptoms on June 23, July 19, August 15, and August 25 in 2005 in the artificial disease nursery. The disease grade of each plant was recorded by the leaf symptoms for BC_2_F_1_ and BC_1_S_1_ on 17 September in 2005 in the experiment field in Anyang. The DI was calculated using all plants scores within the family in each plot, and then the disease index(DI) for per each family was calculated to get the average value using the DI of the three replicates (the phenotype datasets in the Additional file 2). The DI was calculated as follows:

$$ \mathrm{D}\mathrm{I}=\left[\Sigma \left({\mathrm{N}}_{\mathrm{i}}\times \mathrm{i}\right)/\left(\mathrm{N}\times 4\right)\right]\times 100,\ \mathrm{i}=1\hbox{--} 4,\ {\mathrm{N}}_{\mathrm{i}} = \mathrm{n}\mathrm{umber}\;\mathrm{of}\;\mathrm{plants}\;\mathrm{with}\;\mathrm{the}\;\mathrm{grade},\;\mathrm{N}=\mathrm{the}\;\mathrm{total}\;\mathrm{n}\mathrm{umber}\;\mathrm{of}\;\mathrm{plants}\;\mathrm{i}\mathrm{n}\;\mathrm{each}\;\mathrm{family} $$

### DNA extraction, PCR amplification, electrophoresis and map construction

Cotton genomic DNA was extracted from young leaves of the 135 BC_1_F_1_ plants and the two parents as described by Paterson et al. [49]. SSR-PCR was conducted as described by Sun et al. [1] and the PCR products were electrophoresed and silver-stained according to the protocol of Zhang et al. [50]. The genotype datasets are in the Additional file 3. SSR markers were used to construct a genetic map for BC_1_F_1_ population using JoinMap 4.0 [51] and the linkage map was published in 2015 [33].

### QTL analysis

We used the Linkage maps with the phenotypic data of four dates of BC_2_F_1_-NY populations and one date of BC_1_S_1_-FD and BC_2_F_1_-FD populations to identify QTLs for VW resistance. QTLs were analyzed using the composite interval mapping method [52] and Windows QTL Cartographer 2.5 [53], with a walk speed of 1 cM, a window size of 10 cM and 5 background control markers. The statistical significance of the QTLs identified for the trait was determined by running a permutation procedure 1,000 times [54]. Positive additive effects indicated that Hai1 alleles decreased the DI value, increasing the phenotypic values of VW resistance, and negative scores indicated that CCRI 36 decreased the DI value, increasing the values of VW resistance. The QTLs were named as follows: (q + trait abbreviation) + chromosome/linkage group + QTL number. QTLs for the same trait across different generations and dates were considered stable when their confidence intervals overlapped.

### Meta-analysis of QTLs

Recently, Said et al. reported a meta QTL analyses and established CottonQTLdb database containing 2274 QTL for 66 different QTL trait types [34], including 139 QTLs of VW resistance from nine published papers [15, 16, 18, 19, 24–28]. We got the VW resistance QTL mapping information including their names and CI provided by Said et al. [34]. The other 5 studies for VW resistance QTL mapping were also lately reported [13, 17, 21–23], including 65 QTLs of VW resistance. Totally, we used 252 QTLs of VW resistance for Meta-analysis, including 48 QTLs identified in our study.

A meta-analysis of QTLs for VW resistance was performed using Biomercator V3 software [55]. The detailed description for the meta-analysis of QTLs could be found in Said et al. [35, 36]. Hotspots were determined manually. Four or more QTLs in an interval of 25 cM were considered as a consistent QTL region. If there was more than one trait involved in the QTLs, the region is called a QTL cluster. Otherwise, it is called a QTL hotspot for the region involving only one single trait [13].

## Additional files

Additional file 1: The details of QTLs identified using interspecific backcross populations on the map of CCRI36 × Hai1 BC_1_F_1_ and their corresponding hotspots. (XLSX 16 kb)
Additional file 2: The phenotype datasets of BC_2_F_1_ on June 23, July 19, August 15, and August 25 in 2005 in the artificial disease nursery and of BC_2_F_1_ and BC_1_S_1_ on 17 September in 2005 in the field. (XLSX 27 kb)
Additional file 3: The genotype datasets of 135 BC_1_F_1_ plants of CCRI36× Hai1. (XLSX 1247 kb)

## Abbreviations

C: chromosome
cM: CentiMorgan
CSSLs: Chromosome substitution segment substitution lines
DI: Disease index
MAS: Marker assisted selection
QTL: Quantitative trait loci
SSR: Simple sequence repeat
VW: *Verticillium* wilt
